# Neuroplasticity Biomarkers and Neurotransmitter Modulation in Post-Ischemic Stroke Rehabilitation: A Narrative Review

**DOI:** 10.3390/jcm15208091

**Published:** 2026-10-18

**Authors:** Karolina Szymczak-Kumoniewska, Angela Dziedzic, Joanna Saluk, Elżbieta Miller

**Affiliations:** 1 Department of Neurology, Dr. K. Jonscher Muncipal Medical Centre in Lodz, Milionowa 14, 93-113 Łódź, Poland; 2 Department of General Biochemistry, Faculty of Biology and Environmental Protection, University of Lodz, Pomorska 141/143, 90-236 Łódź, Poland; angela.dziedzic@biol.uni.lodz.pl (A.D.); joanna.saluk@biol.uni.lodz.pl (J.S.); 3 Department of Neurological Rehabilitation, Medical University of Lodz, Milionowa 14, 93-113 Łódź, Poland

**Keywords:** stroke, recovery, biomarkers, prognosis, personalized medicine, rehabilitation

## Abstract

**Background/Objectives**: Recovery after stroke is a complex process involving structural and functional reorganization of the nervous system. Neuroplasticity plays a key role in functional recovery, while biomarkers and neurotransmitter systems may reflect or modulate the biological processes underlying rehabilitation and adaptation after stroke. The aim of this review was to summarize the current evidence regarding biomarkers of neuroplasticity and neurotransmitter modulation in post-stroke rehabilitation, with a particular focus on their potential importance for functional recovery and rehabilitation outcomes. **Methods**: This narrative review synthesized evidence identified through searches of PubMed/MEDLINE, Scopus, and Web of Science, supplemented by reference-list screening. The literature was qualitatively analyzed according to the main themes of the review. **Results**: Current data point to BDNF, VEGF, and MMPs as promising biomarkers associated with post-stroke neuroplasticity and functional recovery. Neurotransmitter modulation, particularly involving GABA, glutamate, dopamine, and serotonin, may influence neuronal excitability, synaptic plasticity, and motor learning after stroke. The combination of biomarker assessment and rehabilitation-based modulation of these pathways may provide a more comprehensive approach to understanding and monitoring post-stroke recovery. **Conclusions**: Current evidence supports the role of neuroplasticity-related biomarkers and neurotransmitter modulation as potential tools for understanding biological responses to post-stroke rehabilitation. However, their clinical relevance and usefulness in guiding individualized rehabilitation require further validation in future clinical studies.

## 1. Introduction

Ischemic stroke is a major cause of persistent motor, cognitive, and communication disability. Functional recovery depends not only on lesion size and location but also on the capacity of surviving neural networks to reorganize through synaptic plasticity, changes in cortical excitability, axonal sprouting, angiogenesis, and experience-dependent motor learning. These processes are shaped by interacting molecular and cellular systems, including neurotrophic factors, neurotransmitters, glial cells, and neurovascular remodeling. Rehabilitation provides repeated behavioral input capable of engaging these mechanisms, making biological measures of plasticity potentially relevant for understanding why recovery trajectories and treatment responses differ between patients [1,2,3]. Neurotransmitter systems are particularly relevant because the balance between excitation and inhibition changes dynamically after stroke. Gamma-aminobutyric acid (GABA) and glutamate influence cortical excitability and synaptic plasticity, while dopamine, acetylcholine, and serotonin modulate attention, motivation, motor learning, and network reorganization [1,2,4,5]. In parallel, molecular markers such as brain-derived neurotrophic factor (BDNF), vascular endothelial growth factor (VEGF), nitric oxide synthase 2 (NOS2), and matrix metalloproteinases (MMPs) reflect distinct components of injury, repair, angiogenesis, and remodeling. Previous influential reviews have focused primarily on models of post-stroke cortical reorganization and modulation of brain plasticity, including interhemispheric competition, structural reserve, and non-invasive brain stimulation [6]. The present narrative review takes a complementary translational perspective by integrating molecular biomarkers and neurotransmitter systems with rehabilitation-related modulation and by explicitly distinguishing biological plausibility and prognostic association from evidence of rehabilitation responsiveness and clinical utility. We additionally consider emerging multimodal markers of network reorganization and patient-level modifiers that may influence biomarker interpretation.

## 2. Materials and Methods

This work was designed as a narrative review to provide an integrative, translational synthesis of heterogeneous clinical, preclinical, and mechanistic evidence concerning neuroplasticity biomarkers, neurotransmitter systems, and rehabilitation-related modulation after ischemic stroke. It was not designed as a systematic review or meta-analysis addressing a narrowly predefined intervention question.

Relevant literature published between 2000 and 2026 was identified through searches of PubMed/MEDLINE, Scopus, and Web of Science, conducted in May and June 2026. The search was restricted to English-language publications. The search strategy included combinations of the following keywords: “stroke,” “ischemic stroke,” “biomarker,” “neurotransmitter,” “GABA,” “BDNF,” “VEGF,” “MMP-9,” “microRNA,” “neuroplasticity,” “rehabilitation,” “recovery,” “functional outcome,” and “motor recovery.” Reference lists of relevant publications were also screened to identify additional studies.

An example PubMed/MEDLINE search strategy was: (“stroke” OR “ischemic stroke”) AND (“neuroplasticity” OR “biomarker” OR “BDNF” OR “VEGF” OR “MMP-9” OR “GABA” OR “neurotransmitter”) AND (“rehabilitation” OR “recovery” OR “functional outcome” OR “motor recovery”). Search terms were adapted as appropriate for Scopus and Web of Science.

**Figure 1:**
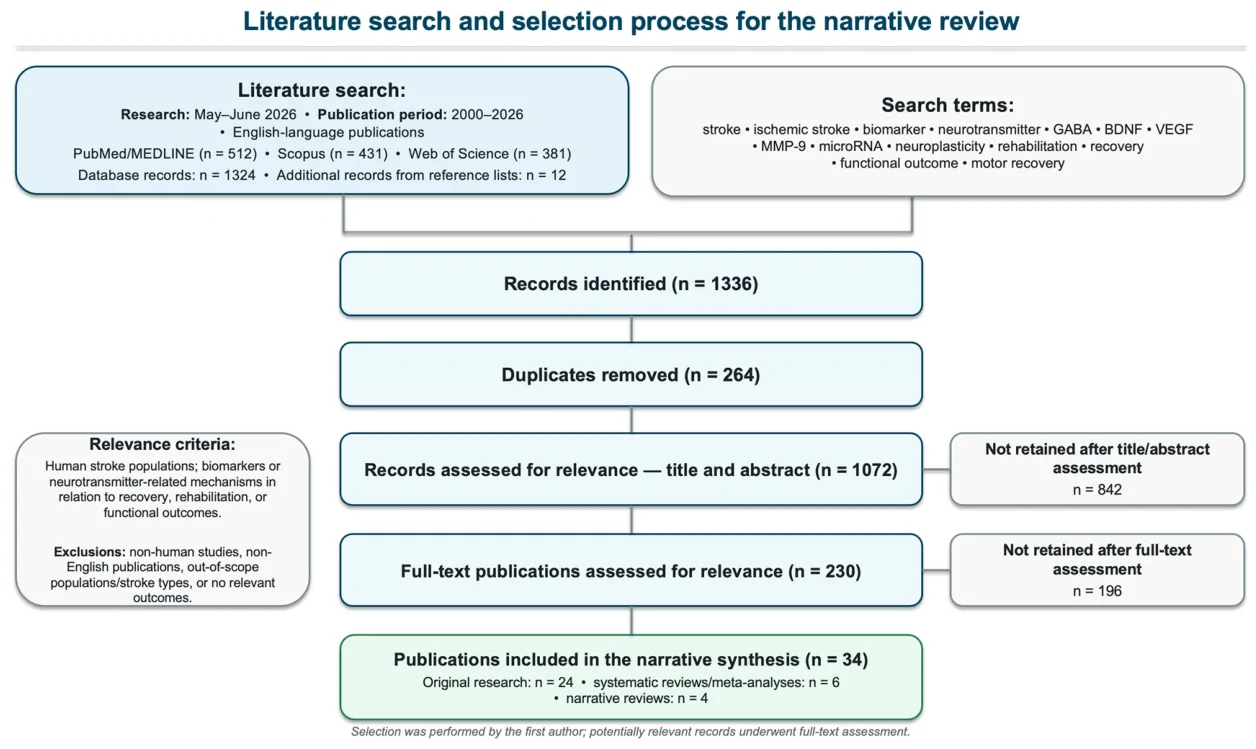
Literature search and selection process for the narrative review.

Titles and abstracts were screened by the first author, followed by full-text assessment of potentially relevant publications. Relevance was determined based on whether studies addressed human stroke populations and biomarkers or neurotransmitter-related mechanisms in relation to recovery, rehabilitation, or functional outcomes. Non-human studies, non-English publications, studies involving inappropriate populations or stroke types, and studies without relevant biomarker/neurotransmitter or recovery outcomes were excluded. The study selection process is presented in Figure 1.

## 3. Results

### 3.1. The Definition and the Role of Neurotransmitters

Under physiological conditions, neurotransmitters shape neuronal networks and control several cellular and synaptic functions. Neurotransmitters are endogenous chemical substances released by synapses responsible for the transmission of signals between nerve cells. Through the process of chemical synaptic transmission, neurotransmitters enable the brain to perform various functions. The process occurring in the human body involves the transmission of neurotransmitters, primarily by releasing them from presynaptic nerve cells to postsynaptic receptors [4]. The basic functionality of each synapse depends heavily on the neurotransmitters released. The most well- known neurotransmitters are: acetylcholine, catecholamines (dopamine and norepinephrine), serotonin, glutamate and gamma-amino- butyric acid (GABA). The main excitatory neurotransmitter in the brain is glutamate. GABA is the main inhibitory substance in the central nervous system. For the proper functioning of the brain, a balance in the transmission of nerve impulses between these two neurotransmitters is essential. Other neurotransmitters, such as acetylcholine, serotonin, and catecholamines have a more modulatory role in the brain. They participate in many neural circuits [4]. During an ischemic stroke, many changes occur around the area of infarction, including changes in plasticity. This process can be considered a new therapeutic target in post-stroke rehabilitation [6].

### 3.2. Neurotransmitters and Neuroplasticity

Neuroplasticity occurs at multiple levels, including cellular, network, and behavioral levels, throughout life. The highest level of neuroplasticity is observed during developmental age, but plasticity also increases during the subacute phase after stroke. This process is associated with the expression of genes and proteins involved in neuronal growth and synapse formation [1]. The most important neurotransmitters in the context of recovery and post-stroke rehabilitation include gamma-aminobutyric acid (GABA), the major inhibitory neurotransmitter, and glutamate, the major excitatory neurotransmitter. GABAergic signaling, including GABA levels, synthesis, and synaptic transmission, changes dynamically after stroke; however, its trajectory is not uniform across patients, brain regions, stroke phases, or measurement modalities. Some studies have reported increased GABA-related measures or inhibitory signaling during the acute phase, which has been interpreted as potentially neuroprotective in the context of glutamatergic excitotoxicity [2]. During subacute and chronic recovery, reduced inhibitory tone has been reported in some cortical regions and may create conditions permissive for experience-dependent plasticity; however, persistent inhibitory abnormalities have also been described. Accordingly, post-stroke GABAergic changes should not be interpreted as a universal transition from increased acute inhibition to decreased inhibition during later recovery. The balance between GABA and glutamate is important, while dopamine, acetylcholine, and serotonin also contribute to motor learning, cortical plasticity, mood, cognition, and network reorganization after stroke [5]. These neurotransmitter-related changes should be interpreted in relation to both the intervention and the outcome measured. Aerobic exercise has been associated with changes in neurotrophic factors and corticomotor excitability, task-specific motor training provides the behavioral context for experience-dependent plasticity, and non-invasive brain stimulation can modify measures of cortical excitability and inhibitory/facilitatory network dynamics. Biological or neurophysiological modulation, however, should not be assumed to mediate functional recovery unless it is directly associated with standardized motor or functional outcomes. Thus, mechanistic effects and clinical benefit should be distinguished consistently (summarized in Table 1).

The trajectory of GABAergic inhibition after stroke is not uniform. Although reduced inhibitory tone in the affected hemisphere has been interpreted as permissive for plasticity, transcranial magnetic stimulation studies have also demonstrated persistent inhibitory abnormalities during early and late recovery. Manganotti et al. showed that motor cortical disinhibition evolves over time and is related to clinical recovery. [7] Thus, recovery cannot be reduced to a simple monotonic decrease in GABAergic tone: inhibitory balance is dynamic, region- and phase-dependent, and interacts with structural reserve and motor training. A single increase/decrease label is therefore insufficient to characterize GABA as a rehabilitation biomarker.

### 3.3. The Definition of Biomarkers and Their Role in Neuroplasticity

In addition to neurotransmitter signaling, neuroplasticity after stroke is also regulated by a number of molecular factors involved in neuronal growth, angiogenesis, and tissue remodeling. These processes are represented by specific biological indicators that can be used to assess brain regeneration and response to therapy. Biomarkers are measurable indicators of biological processes, pathological states, or responses to therapeutic interventions. Naylor, in providing the classic definition of a biomarker, assumed that it should be objectively measured and evaluated as a marker of physiological and pathological processes, as well as an indicator of response to therapeutic intervention. Biomarkers are widely used in disease risk assessment, diagnostic purposes, and prediction of treatment outcomes. However, for a biomarker to be clinically useful, it must meet several key requirements: it must be sensitive and specific, the collection process must be safe and noninvasive, and the measurements must be reproducible. Furthermore, the assay methods used should be rapid, cost-effective, and non-interfering with ongoing treatment [3]. Biomarkers of recovery from ischemic stroke can provide valuable information regarding the course of recovery and likely response to treatment. The literature distinguishes two basic types of such biomarkers: prognostic, which assess future clinical outcomes regardless of the therapy used, and predictive, which identify patients most likely to benefit from specific therapeutic interventions. Effective use of biomarkers allows for patient phenotyping, monitoring disease progression and response to treatment and consequently, drug discovery with increasing precision [8].

### 3.4. Biomarkers of Stroke Recovery

**Figure 2:**
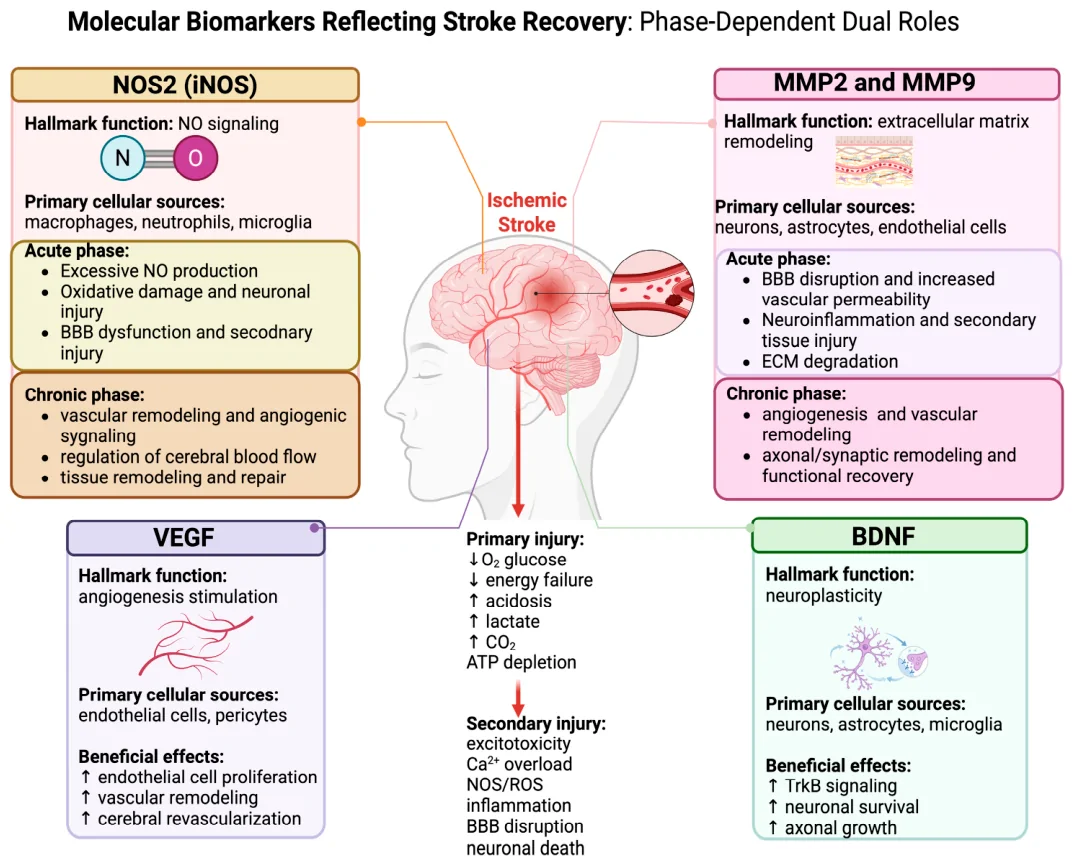
Molecular biomarkers reflecting key biological processes involved in post-stroke recovery. Ischemic stroke initiates primary and secondary injury cascades that trigger distinct regenerative and pathological responses. NOS2 (iNOS), VEGF, BDNF, and MMP-2/MMP-9 represent biomarkers associated with major mechanisms of post-stroke recovery, including NO signalling, angiogenesis, neuroplasticity, and extracellular matrix remodeling. The red arrows illustrate the progression from primary ischemic injury, characterized by oxygen and glucose deprivation and subsequent metabolic disturbances, to secondary injury involving excitotoxicity, oxidative stress, neuroinflammation, BBB disruption, and neuronal death. Created in BioRender. Dziedzic, A. (2026) https://BioRender.com/rq251y4. Abbreviations: BBB, blood–brain barrier; BDNF, brain-derived neurotrophic factor; Ca^2+^, calcium ions; CO_2_, carbon dioxide; ECM, extracellular matrix; MMP-2, matrix metalloproteinase-2; MMP-9, matrix metalloproteinase-9; NO, nitric oxide; NOS2 (iNOS), inducible nitric oxide synthase; O_2_, oxygen; ROS, reactive oxygen species; TrkB, tropomyosin receptor kinase B; VEGF, vascular endothelial growth factor.

The pathophysiology of ischemic stroke involves the activation of numerous molecular and cellular pathways, such as excitotoxicity, oxidative stress, inflammatory response, angiogenesis, extracellular matrix remodeling, and neuroplastic processes, which largely determine the regenerative potential of the central nervous system and the effects of functional recovery. Due to this cascade of changes the literature distinguishes biomarkers related to angiogenesis, oxidative stress, extracellular matrix remodeling and neuroplasticity, as summarized in Table 2. Figure 2 illustrates molecular biomarkers reflecting key biological processes involved in post-stroke recovery, highlighting their dual, phase-dependent roles in the pathophysiology of ischemic stroke. 

#### 3.4.1. NOS2

Cerebral ischemia produces energy failure, membrane depolarization, ionic imbalance, excessive intracellular Ca^2+^ accumulation, mitochondrial dysfunction, oxidative and nitrosative stress, and inflammatory activation [9]. Nitric oxide (NO) signaling is strongly time-, concentration-, and cell-dependent. Following ischemia, NOS isoforms contribute differently to vascular regulation, inflammatory responses, and tissue injury. NOS2 (iNOS) is induced in inflammatory and glial cells and can generate sustained NO production; in an oxidative environment, NO may react with superoxide to form reactive nitrogen species that contribute to cellular injury [10]. At the same time, NO-related pathways may participate in vascular responses and later repair processes. Therefore, NOS2 should not be interpreted as a specific marker of rehabilitation-induced neuroplasticity. Its potential relevance depends on stroke phase and biological context, and current evidence primarily supports its role as a marker/mechanism of inflammatory and oxidative injury rather than as a validated rehabilitation-response biomarker [10,11].

#### 3.4.2. VEGF

Angiogenesis plays a crucial role in neuronal reorganization and regeneration after stroke. Cerebral ischemia is a potent stimulus inducing angiogenesis as an adaptive response to hypoxia. The most potent angiogenic factor induced by hypoxia in response to ischemic stroke is VEGF. It is secreted by endothelial cells and pericytes. VEGF was discovered in 1983. VEGF acts through the tyrosine kinase receptors VEGFR-1 (Flt-1) and VEGFR-2 (KDR/Flk-1). It is a specific mitogen for endothelial cells. In the context of ischemic stroke, its action is associated with increased neovascularization and collateral vessel formation in ischemic areas. Studies have shown that serum VEGF concentrations are elevated in stroke patients and persist for approximately 3 months after ischemic stroke [12]. VEGF is regulated by other growth factors in the first hours after stroke and plays a key role in vascular remodeling in the injured brain. Therefore, it likely exerts a beneficial effect in ischemic stroke. It may be a potential method for cerebral revascularization [13].

#### 3.4.3. BDNF

Low serum BDNF levels in the acute phase after stroke have been associated with poorer functional outcomes at 90 days [14]. Similarly, a meta-analysis by Karantali et al. demonstrated significantly lower BDNF levels in patients with acute ischemic stroke compared to healthy controls. The authors also observed a negative correlation between BDNF levels and the severity of neurological deficit assessed using the NIHSS scale, indicating that lower BDNF levels are associated with greater neurological severity in acute stroke. Furthermore, preclinical studies have investigated strategies to enhance BDNF/TrkB signaling in experimental ischemic stroke. Wang et al. reported that combined administration of intramuscular AAV-BDNF and intranasal AAV-TrkB promoted neurological recovery in stroke rats, which was associated with enhanced corticospinal synaptic connections [15]. Brain-derived neurotrophic factor (BDNF) belongs to the neurotrophin family of growth factors, which also includes nerve growth factor (NGF), neurotrophin-3 (NT-3), NT4/5, and NT-6. It is one of the neurotrophic factors that demonstrates neuroprotective effects under adverse conditions such as glutamatergic stimulation, cerebral ischemia, hypoglycemia, and neurotoxicity. Upon binding to receptor tyrosine kinase B (TrkB), BDNF activates several signaling pathways: Akt/PKB, phosphoinositide 3-kinase (PI3K), and insulin receptor substrate 1 or 2 (IRS1/2), promoting protein synthesis, cell survival, dendritic maturation, axonal growth, synaptic plasticity, stress resistance, and therapeutic neovascularization. Both BDNF protein and its mRNA are widely distributed in the central nervous system. Its presence has been demonstrated in numerous brain structures, including the cerebral cortex, hippocampus, olfactory bulb, basal forebrain, midbrain, hypothalamus, brainstem, and spinal cord. This widespread distribution of BDNF underscores its importance in regulating neuronal function, learning and memory processes and neuroplasticity mechanisms after stroke. BDNF levels are decreased in many neurodegenerative diseases, such as Parkinson’s disease (PD), multiple sclerosis (MS), and Huntington’s disease [16]. Due to its broad spectrum of neurotrophic effects and changes in its concentration during recovery, BDNF is considered a promising biomarker of recovery from stroke. A study by Lasek-Bal et al. showed that lower BDNF levels in the acute phase of ischemic stroke correlated with greater severity of neurological deficit assessed using the NIHSS scale. Reduced BDNF levels were also associated with poorer functional prognosis during follow-up, supporting the potential prognostic relevance of BDNF in human ischemic stroke [14]. The meta-analysis by Karantali et al. reported lower serum BDNF levels in patients with acute ischemic stroke than in healthy controls and an association between BDNF levels and neurological deficit assessed using the NIHSS [17]. Neurological rehabilitation may influence BDNF-related signaling; however, rehabilitation-induced changes in BDNF should be distinguished from prognostic associations observed in the acute phase. Preclinical BDNF-targeted strategies remain experimental and should not be interpreted as established clinical rehabilitation interventions. Exercise-related changes in BDNF in stroke survivors are discussed separately below [18].

#### 3.4.4. MMP-2, MMP-9

Matrix metalloproteinases (MMPs) are zinc-dependent endopeptidases involved in extracellular matrix remodeling, vascular biology, inflammation, and tissue repair. Their effects after ischemic stroke are strongly phase-dependent. In the acute phase, increased MMP-9 activity is associated with extracellular matrix degradation, blood–brain barrier disruption, hemorrhagic transformation, and worse clinical outcomes [19]. In later phases, MMP activity may also participate in extracellular matrix remodeling, angiogenesis, and synaptic reorganization. MMP-2 contributes to neurovascular remodeling and is expressed in neurons, astrocytes, endothelial cells, and vascular smooth muscle cells. Elevated MMP-2 and MMP-9 concentrations after stroke therefore cannot be interpreted simply as evidence of beneficial neuroplasticity. Depending on timing and context, they may reflect tissue injury, vascular remodeling, or subsequent repair. Associations of MMP-2 with atrial fibrillation and vascular remodeling are relevant to stroke biology but do not establish MMP-2 as a marker of rehabilitation response [20]. Accordingly, MMP-2 and MMP-9 should currently be regarded as biologically relevant, phase-dependent candidate markers rather than validated tools for monitoring rehabilitation.

**Table 2:** Current evidence and potential rehabilitation relevance of selected biomarkers in post-ischemic stroke recovery.

Biomarker	Main Biological Role After Ischemic Stroke	Current Evidence in Relation to Recovery/Rehabilitation	Current Clinical Interpretation
BDNF	Neurotrophic signaling, neuronal survival, synaptic plasticity and motor learning.	Lower circulating BDNF in the acute phase has been associated with greater neurological deficit and poorer functional outcome [14,17]. Exercise and rehabilitation may influence BDNF-related signaling, supporting its biological relevance to neuroplasticity [18].	Among the biomarkers discussed, BDNF has the most direct relationship with neuroplasticity; however, it is not validated for routine monitoring of rehabilitation response.
VEGF	Hypoxia-related angiogenesis, neovascularization and neurovascular remodeling.	VEGF levels change after ischemic stroke and may remain elevated during recovery. Exercise-related changes in angiogenic pathways provide a plausible link with rehabilitation, but available evidence largely reflects vascular remodeling and recovery biology rather than a specific rehabilitation response [12,13].	Biologically relevant to recovery, but evidence is insufficient to use VEGF as a validated rehabilitation-response biomarker.
NOS2 (iNOS)	Inflammatory and oxidative/nitrosative signaling with time- and context-dependent effects.	NOS2 induction after cerebral ischemia is mainly associated with inflammatory and oxidative injury mechanisms. Its relationship with later repair is complex and phase-dependent [10,11].	Not a specific biomarker of rehabilitation-induced neuroplasticity; current evidence mainly supports a role in injury-related biology.
MMP-9	Extracellular-matrix degradation and remodeling; acute blood–brain barrier disruption, with possible later remodeling effects.	Acute elevation is associated mainly with tissue injury, hemorrhagic transformation and poorer prognosis. Later MMP activity may contribute to extracellular-matrix remodeling, but direct evidence of responsiveness to rehabilitation is limited [19].	Predominantly an injury/prognostic marker in the acute phase; its role in monitoring rehabilitation remains unproven.
MMP-2	Extracellular-matrix and neurovascular remodeling.	MMP-2 is involved in vascular and tissue remodeling and may be elevated after ischemic stroke. Associations with vascular remodeling and atrial fibrillation are relevant to stroke biology but do not establish a rehabilitation-response biomarker [20].	Potential mechanistic relevance to recovery, but not validated as a marker of rehabilitation response.

### 3.5. Multimodal Biomarkers of Network Reorganization

Molecular biomarkers provide only one level of information about post-stroke plasticity. Multimodal approaches may better capture network reorganization by combining biological signals with neurophysiology, neuroimaging, and quantitative behavior. Quantitative electroencephalography (EEG) measures such as the pairwise-derived Brain Symmetry Index and the delta–theta/alpha–beta ratio have been associated with later functional status after ischemic stroke [21]. Integration of EEG with upper-limb kinematics has also shown relationships among clinical motor scores, movement duration, smoothness, velocity peaks, and electrophysiological measures at four weeks after stroke [22]. Similar frameworks can incorporate structural and functional magnetic resonance imaging, diffusion-based measures of corticospinal tract integrity, and connectivity metrics. At present, these measures should be considered candidate proxies of network-level plasticity rather than validated tools for selecting rehabilitation treatment.

### 3.6. Non-Invasive Brain Stimulation and Neuroplasticity

Non-invasive brain stimulation (NIBS), including repetitive transcranial magnetic stimulation (rTMS) and transcranial electrical stimulation (tES), can modulate cortical excitability and network dynamics and therefore provides both a mechanistic probe and a potential adjunct to rehabilitation. Earlier models emphasized correction of interhemispheric imbalance, but this framework is not applicable to all patients; effects depend on lesion characteristics, residual structural reserve, stimulation target, timing, and concurrent behavioral training [6]. Contemporary approaches increasingly use network-informed, state-dependent, multifocal, and individualized targeting. Recent evidence supports biological and therapeutic potential in stroke, but treatment effects remain heterogeneous and large controlled trials are still needed before broad clinical implementation [23].

### 3.7. Glial Contributions to Post-Stroke Plasticity

Post-stroke plasticity is not exclusively neuronal. Astrocytes regulate extracellular glutamate and GABA homeostasis, metabolic support, neurovascular coupling, synaptogenesis, and trophic signaling, while reactive astrocytes can have both protective and maladaptive effects depending on timing and context [24]. Microglia likewise contribute to inflammation, synaptic pruning, neurogenesis, axonal remodeling, and network rewiring [25]. Neurotransmitter dynamics after stroke therefore partly reflect neuron–glia interactions rather than neuronal signaling alone, helping to explain why the same pathway may be detrimental during acute injury yet contribute to remodeling during later recovery.

### 3.8. Emerging Molecular Biomarkers

The biomarker landscape also extends beyond proteins. Circulating microRNAs (miRNAs) and extracellular-vesicle-derived miRNAs are being investigated as markers of motor and cognitive recovery after ischemic stroke. A systematic review and meta-analysis identified multiple circulating miRNAs associated with functional recovery, although heterogeneity and limited external validation remain important barriers [26]. Extracellular vesicles, including exosomes, may also participate directly in intercellular signaling and neurovascular remodeling by transferring miRNAs and other cargo, but much of the therapeutic evidence remains preclinical [27]. Metabolic mediators such as lactate are also of interest because neuronal–glial metabolic coupling can influence activity-dependent plasticity; however, rehabilitation-specific clinical evidence is currently less mature. These emerging markers broaden the mechanistic framework but should not be considered equivalent in evidentiary strength to clinically validated biomarkers.

## 4. Discussion

The most relevant finding of this narrative review is that post-stroke recovery cannot be represented by a single molecular marker or neurotransmitter direction. A coherent interpretation requires a temporal and multilevel framework: acute injury-related processes (including NOS2 and early MMP activity), subsequent vascular and extracellular-matrix remodeling (including VEGF and later MMP effects), neurotrophic and synaptic plasticity (particularly BDNF and excitation–inhibition balance), and network-level reorganization measurable with neurophysiology, imaging, and quantitative behavior. These processes overlap rather than occur in strictly separated phases. Accordingly, the clinical value of a candidate biomarker depends on when it is measured, what biological compartment it represents, and whether it predicts spontaneous recovery or a differential response to a defined rehabilitation intervention.

Early, intensive rehabilitation focused on specific tasks supports adaptive neuroplasticity and limits the development of unfavorable maladaptive changes. One of the most well-known factors promoting neuroplasticity is aerobic exercise, which can go beyond improving cardiorespiratory fitness [28]. A growing body of research suggests that aerobic exercise can increase the expression of neurotrophic factors, particularly BDNF, as well as IGF-1 and VEGF, thereby stimulating neuroplastic processes. BDNF is of particular importance, playing a key role in neurogenesis, synaptic reorganization, and motor learning. The results of a meta-analysis by Ashcroft et al. indicate that aerobic exercise is the preferred form of exercise that optimally increases BDNF levels in adults after stroke. Both a single session and a long-term aerobic training program can lead to an increase in BDNF levels. Exercise intensity appears to be a key factor, with the greatest effects observed after high-intensity exercise [18]. Increasing VEGF expression through aerobic training plays a significant role in angiogenesis and improving metabolic conditions for neural tissue regeneration, thereby also supporting neuroplasticity. In addition to increasing neurotrophin expression, aerobic training is also believed to modulate neurotransmitters systems, the most important of which is the balance between GABA and glutamate. These phenomena are particularly important in the context of motor learning, which forms the basis of effective neurological rehabilitation. The literature suggests that aerobic training can serve as “aerobic priming,” an intervention that prepares the nervous system for more effective motor learning. It can temporarily increase corticomotor excitability and promote neuroplasticity, potentially increasing the effectiveness of subsequent task-specific training [28]. Increasing evidence suggests that the effects of post-stroke rehabilitation are associated not only with functional improvements observed at the clinical level but also with the activation of biological mechanisms underlying neuroplasticity. That’s why a lot of attention is given to biomarkers that reflect neurogenesis, angiogenesis, and neural network remodeling. Among the most widely studied biomarkers are BDNF, VEGF, matrix metalloproteinases and selected neurotransmitter systems, whose expression and activity may reflect the brain’s capacity for functional recovery and responsiveness to rehabilitation interventions. Although several biomarkers have shown potential utility in assessing post-stroke recovery, none has proven capable of independently capturing the full spectrum of biological processes involved. Functional improvement results from the interaction of multiple molecular and cellular pathways, including neurogenesis, synaptic remodeling, angiogenesis, inflammation, and modulation of excitatory inhibitors and neurotransmission. Therefore, it is unlikely that a single biological marker can adequately capture the complexity of these processes. Considering the multifactorial nature of post-stroke rehabilitation, looking at a combination of biomarkers that reflect neuroplasticity, angiogenesis, inflammation, and neuron repair can give a clearer picture of the regenerative processes. Such a multimarker approach is biologically plausible and may become useful for stratifying recovery phenotypes, but its clinical validity and utility in rehabilitation have not yet been established. Despite significant advances in elucidating the biological mechanisms of post-stroke recovery, further investigations are needed to develop standardized biomarker assessment protocols and to define their clinical applicability. In particular, future research should aim to validate multimarker panels reflecting distinct components of neuroplasticity and to assess their usefulness in supporting personalized rehabilitation approaches. Taken together, rehabilitation remains the central pillar of post-stroke recovery. Its efficacy appears to be closely linked to the activation of neuroplasticity-related mechanisms, including neurotrophic, angiogenic, and neurotransmitter pathways. Advancing knowledge in this area may eventually support biomarker-informed rehabilitation; however, evidence of analytical validity, clinical validity, actionable thresholds, responsiveness to rehabilitation, and improved outcomes from biomarker-guided treatment is still required [3,9].

To interpret rehabilitation-related modulation more explicitly, representative interventions should be linked to both measured biological effects and functional outcomes. In the studies discussed above, aerobic exercise has been evaluated using neurotrophic/angiogenic markers such as BDNF and VEGF and neurophysiological measures of corticomotor excitability, while task-specific training is assessed primarily through motor learning and functional performance. Non-invasive brain stimulation provides measurable indices of cortical excitability and inhibitory/facilitatory network dynamics and may be combined with behavioral training. However, parallel changes in a biological marker and a clinical outcome do not by themselves establish mediation. Stronger mechanistic inference requires studies that directly relate within-patient biological or neurophysiological change to standardized motor or functional improvement after a defined rehabilitation intervention [18,23,28].

### 4.1. Patient-Level Modifiers of Recovery and Biomarker Interpretation

Sex may modify the clinical context in which recovery biomarkers are interpreted. In a 2024 registry study of acute cardioembolic ischemic stroke, women differed from men in risk-factor profile, clinical presentation, stroke severity, and early outcome [29]. However, sex-stratified evidence linking circulating neuroplasticity biomarkers or neurotransmitter measures specifically to rehabilitation response remains limited. Because the present work is a narrative review rather than a prespecified systematic protocol, sex was not used as an eligibility or stratification criterion. Future rehabilitation studies should prospectively report sex-specific biomarker trajectories and test sex-by-treatment interactions.

Stroke subtype and lesion topography are also important sources of biological and functional heterogeneity. Pure motor stroke is most commonly associated with lacunar infarction and has a more favorable short-term functional profile than non-lacunar stroke; in the study by Arboix et al., 85% of pure motor strokes were lacunar and outcomes were better than in non-lacunar stroke [30]. Lesion location may be at least as important as lesion volume for motor recovery: damage involving the internal capsule, corona radiata, corticospinal pathways, putamen, and related motor networks has been associated with motor impairment and functional outcome [31,32]. Accordingly, biomarker and neurotransmitter findings should not be assumed to have identical meaning in a small subcortical lacunar pure motor stroke and in a larger cortical or cortico-subcortical infarction with hemiplegia. Direct comparative biomarker studies across these phenotypes are still lacking.

Very old patients represent another clinically important subgroup. Studies of patients aged 85 years or older show a different demographic and vascular-risk profile, with a high proportion of women and greater prevalence of atrial fibrillation and cardiac comorbidity, together with more severe stroke and poorer functional outcomes than in younger cohorts [33,34]. These differences may influence baseline biomarker concentrations, inflammatory and vascular responses, neuroplastic potential, treatment tolerance, and rehabilitation trajectories. Dedicated studies in very old patients are therefore needed before biomarker thresholds or rehabilitation-response models derived from younger populations can be generalized to this age group.

### 4.2. Limitations of Current Biomarker Evidence

Current biomarker evidence in post-stroke rehabilitation is limited by substantial heterogeneity in study design, stroke phase, biological sample, assay methodology, timing of measurement, lesion characteristics, medication exposure, age and comorbidities, and rehabilitation type and dose. Peripheral blood concentrations may not directly reflect central nervous system processes, and temporal variability is particularly important for phase-dependent markers such as NOS2 and MMPs. Standardized cut-offs and clinically actionable thresholds are lacking. Most available studies demonstrate biological plausibility or prognostic association rather than predictive value for a specific rehabilitation intervention. Consequently, none of the biomarkers discussed is currently sufficiently validated to guide routine individualized rehabilitation. In addition, because this article is a narrative review, it does not provide the exhaustive study identification, formal risk-of-bias assessment, or quantitative evidence synthesis expected from a PRISMA-based systematic review or an umbrella review. These approaches would be valuable for future focused questions when sufficiently homogeneous systematic reviews or primary studies are available. An additional limitation is the narrative design of the present review: study identification and synthesis were not performed according to a systematic-review protocol, and no formal risk-of-bias assessment or quantitative evidence grading was undertaken. Moreover, sex, age, lacunar versus non-lacunar stroke, and lesion topography were not prespecified stratification variables, which limits subgroup-specific conclusions.

### 4.3. Future Research Directions

Future research should prioritize prospective, rehabilitation-specific cohorts with repeated biomarker measurements linked to standardized functional outcomes. Prespecified analyses should address sex, age, stroke subtype, lesion topography and corticospinal tract involvement, as well as rehabilitation type, intensity, and dose. Particularly informative designs would compare lacunar pure motor stroke with non-lacunar motor stroke, include very old patients, integrate blood biomarkers with neuroimaging or neurophysiological measures, and test whether biomarker changes predict differential response to a defined rehabilitation intervention rather than spontaneous recovery alone.

## 5. Conclusions

In summary, rehabilitation remains a key element of recovery after ischemic stroke. Physical exercise, particularly aerobic exercise, can influence neurotrophic, angiogenic, and neurotransmitter-related pathways and may contribute to measurable biological responses associated with recovery. However, the biomarkers discussed differ substantially in biological meaning: some primarily reflect injury or prognosis, whereas others have a more direct relationship with neuroplasticity. Current evidence does not establish validated predictive biomarkers of rehabilitation response, standardized clinical thresholds, or clinical utility for biomarker-guided rehabilitation. Therefore, none of the biomarkers discussed are currently ready to guide routine individualized post-stroke rehabilitation. Prospective, rehabilitation-specific studies with standardized measurement protocols are required before these approaches can be translated into clinical practice. Future systematic reviews or umbrella reviews may complement this broad narrative synthesis by addressing narrower, predefined questions concerning individual biomarkers, specific rehabilitation interventions, or defined clinical outcomes.

## Figures and Tables

**Table 1:** Changes in major neurotransmitter systems after stroke and their potential role in neuroplasticity and functional recovery.

Neurotransmitter	Stroke Phase	Change in Level/Activity	Function/Impact on Neuroplasticity	References
GABA	Acute (0–7 days)	Reported increase in GABA-related measures in some acute-phase studies; findings are method- and region-dependent	May contribute to limiting excitotoxicity; proposed neuroprotective role	[1,2]
	Subacute/Chronic	Reduced inhibitory tone reported in some cortical regions; persistent inhibitory abnormalities are also described	May create conditions permissive for experience-dependent plasticity; relationship with functional recovery is not uniform	[1,2]
Glutamate	Acute	Acute glutamatergic/excitatory excess may occur	Excessive signaling may contribute to excitotoxicity	[2,5]
	Subacute/Chronic	Later changes vary by region, phase, and measurement method	Physiological glutamatergic signaling contributes to synaptic plasticity and learning	[5]
Dopamine	Subacute/Chronic	Modulation of dopaminergic activity; direction and magnitude vary by context	Involved in motor learning and network reorganization; functional effects depend on context	[5]
Acetylcholine	Subacute/Chronic	Modulation reported; direction depends on phase, region, and measurement approach	Supports attention, learning, and cortical plasticity; clinical relevance requires outcome-specific evidence	[5]
Serotonin	Subacute/Chronic	Modulation reported; direction depends on phase, region, and measurement approach	May influence plasticity, mood, and recovery-related behavior; functional effects are context-dependent	[5]

## Data Availability

No new data were created or analyzed in this study. Data sharing is not applicable to this article.

## Abbreviations

The following abbreviations are used in this manuscript:

DALYs	disability-adjusted life years
rt-PA	recombinant tissue plasminogen activator
EVT	endovascular thrombectomy
BDNF	brain-derived neurotrophic factor
VEGF	vascular endothelial growth factor
MMPs	matrix metalloproteinases
GABA	gamma-aminobutyric acid
NOS2	nitric oxide synthase 2
iNOS	inducible nitric oxide synthase
NO	nitric oxide
MMP-2	matrix metalloproteinase-2
MMP-9	matrix metalloproteinase-9
